# Fine root dynamics and its contribution to soil organic carbon stocks with *Caragana intermedia* plantation development in alpine sandy land

**DOI:** 10.3389/fpls.2023.1093678

**Published:** 2023-03-09

**Authors:** Qingxue Li, Zhiqing Jia, Lingxianzi He, Xuebin Zhao, Henghua Yang

**Affiliations:** ^1^ Institute of Ecological Conservation and Restoration, Chinese Academy of Forestry, Beijing, China; ^2^ Qinghai Gonghe Desert Ecosystem Research Station, China Terrestrial Ecosystem Research Network, Qinghai, China; ^3^ Research Institute of Forestry, Chinese Academy of Forestry, Beijing, China

**Keywords:** desert ecosystem, shrub plantation, fine root dynamics, soil organic carbon, Tibetan Plateau

## Abstract

Shrubs are the main species in desert ecosystems. Better understanding shrubs fine root dynamics and its contribution to soil organic carbon (SOC) stocks can improve the accuracy of carbon sequestration estimation and provide basic data for the calculation of carbon sequestration potential. The ingrowth core method was used to investigate the fine root (< 1 mm in diameter) dynamics of *Caragana intermedia* Kuang et H. C. Fu plantation with different age (4-, 6-, 11-, 17-, and 31-year-old) in Gonghe Basin of the Tibetan Plateau, and the annual fine root mortality was used for calculation the annual carbon input to SOC pool. The results showed that fine root biomass, production, and mortality first increased and then decreased as the plantation age increased. Fine root biomass peaked in 17-year-old plantation, production and mortality peaked in 6-year-old plantation, and turnover rate of 4- and 6-year-old plantations were significantly higher than other plantations. Fine root production and mortality were negative correlated with soil nutrients at depth of 0–20 and 20–40 cm. The variation range of carbon input by fine root mortality across different plantation age at 0–60 cm soil depth was 0.54–0.85 Mg ha^-1^ year^-1^, accounting for 2.40–7.54% of the SOC stocks. *C. intermedia* plantation has a strong carbon sequestration potential from long time scale. Fine roots regenerate faster in young stands and lower soil nutrients environment. Our results suggest that the influences of plantation age and soil depth should be taken into account when calculating the contribution of fine root to SOC stocks in desert ecosystems.

## 1. Introduction

The carbon storage in arid and semi-arid sandy areas accounts for a small proportion of the total organic carbon storage in terrestrial ecosystems, but due to its wide distribution, it has a very important impact on the global carbon cycle (McCormack et al., 2013; Lai et al., 2017). Under the global trend of climate change, the implementation of vegetation restoration measures for desert ecosystems in arid and semi-arid areas can restore degraded land and increasing the carbon sequestration potential. Enhanced desertification control plays an important role in the global carbon cycle and in addressing the rise of CO_2_ in the atmosphere. Shrub is an important vegetation type for ecosystem restoration in desert areas. As global climate change becomes more and more serious, people have paid more attention to the shrubs that are widely distributed in arid and semi-arid areas in recent years (Lai et al., 2016; Liu et al., 2021; Nie et al., 2022).

Soil carbon pool is the most active component of desert ecosystem carbon pool in arid and semi-arid regions, which plays an important role in regulating regional ecosystem carbon cycle and mitigating global climate change. Fine root, which connects plant and soil, is not only the main component of underground ecosystem carbon pool but also an indispensable part of the carbon cycle. Moreover, the contribution rate of fine root to soil carbon storage may be much greater than that of above-ground litter (Majdi et al., 2008). Previous studies indicated that approximately 30–80% of SOC content is provided through the rapid turnover and decomposition of fine root (Ruess et al., 2003). The production and turnover of fine root are very susceptible to genetic and environmental factors, such as stand age, soil temperature, soil water content, soil chemical, and physical properties (Batkhuu et al., 2021; Jiang et al., 2021; Song et al., 2022). However, due to the invisibility of fine roots, it is difficult to sample, observe, and analyze, resulting in researches on the contribution of fine root to soil carbon pool are much less than those on aboveground parts (Finér et al., 2011). At present, although a large number of studies have estimated the contribution rate of fine root turnover and decomposition to SOC storage, most of them focus on forest, grassland, and farmland ecosystems, and few studies have focused on desert ecosystems with large area in arid and semi-arid areas (Ruess et al., 2003; Lai et al., 2017). Therefore, the study of fine root dynamics is essential for further understanding of their role as a source of litter and SOC storage in soil for desert ecosystems in arid and semi-arid regions.

*Caragana* spp. plants are perennial leguminous shrubs, distributed all over the world, most of which are important constructive species in arid and semi-arid regions of Eurasia (Ji et al., 2019). *Caragana* spp. plants have strong cold and drought-resistant, barren-tolerant ability, and regeneration activity. They play an important role in wind prevention and sand fixation, soil and water conservation, and ecosystem degradation prevention in arid and semi-arid desert areas of northern China (Ji et al., 2019). The Gonghe Basin is located in the northeast of the Qinghai-Tibet Plateau; it is an important ecological barrier for China. The ecological environment of this region is fragile, and it is one of the regions seriously affected by desertification. Vegetation restoration measures have been carried out in the Gonghe Basin since 1958 and have obtained good results. *Caragana intermedia* Kuang et H. C. Fu is the main species for vegetation restoration on moving sand dunes in this region. At present, *C. intermedia* plantations are widely distributed on sand dunes. Better understanding the fine root dynamics and its contribution to SOC stocks during *C. intermedia* plantation development in alpine sandy land can improve the accuracy of carbon sequestration estimation and provide basic data for studying the carbon sequestration potential of desert ecosystem on a larger scale. Therefore, we performed a sampling campaign in different age of *C. intermedia* plantations to answer the following scientific questions: (1) How fine root biomass, production, mortality, and turnover rate changes with the plantation age? (2) What are the main factors influenced fine roots dynamics? (3) How much does the fine root mortalities contribute to SOC accumulation?

## 2. Materials and methods

### 2.1 Study area

This study was conducted at the Desertification Combating Experimental Site of the Qinghai Gonghe Desert Ecosystem Research Station (99°45′–100°30′E, 36°03′–36°40′N, and altitude 2871 m). The climate belongs to the transition zone of alpine arid desert and semi-arid grassland. The mean annual air temperature is 2.4°C. The mean annual precipitation is 246.3 mm, and the mean annual potential evaporation is 1716.7 mm. The mean annual frost-free period is 91 days, and the total solar radiation is 6631.69 MJ·m^-2^·a^-1^. The mean annual number of windy days is 50.6 days, and the maximum wind speed reaches 40 m·s^-1^. The main vegetation type of the study area is sand-fixing plantations, dominated by the tree species *Populus cathayana* Rehd and *Populus simonii* Carr. and the shrub species *C. intermedia*, *Caragana korshinskii* Kom., *Salix cheilophila* Schneid., *Salix psammophila* C. Wang et Chang Y. Yang, and *Hippophae rhamnoides* Linn. The zonal soil is chestnut soil, and the azonal soils are eolian sandy soil, meadow soil, and bog soil (Li et al., 2019). The soil of *C. intermedia* plantations in this study is eolian sandy soil.

### 2.2 Experimental design and sampling

**Figure 1:**
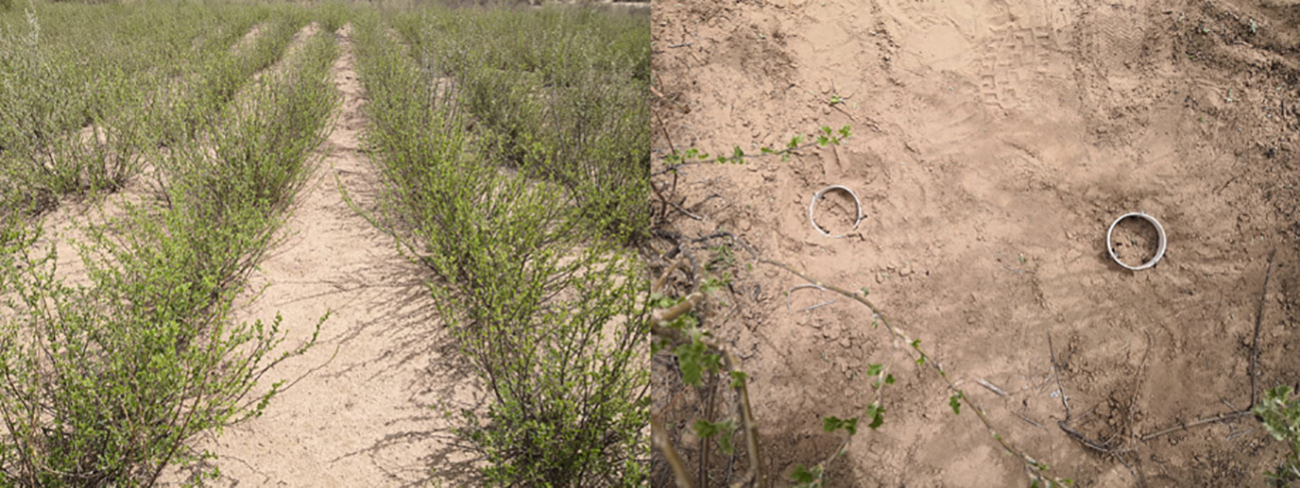
The *C. intermedia* plantation plot and sampling points.

The ingrowth core method can directly determine fine root growth (Yuan and Chen, 2012). Therefore, in this study, the ingrowth core method was used to investigate the fine root biomass, annual fine root production, mortality, and turnover rate. The study was carried out in May 2018. Three replicate plots (20 m × 20 m) were established for sampling in 4-, 6-, 11-, 17-, and 31-year-old *C. intermedia* plantations; the distance between each plot was greater than 20 m. All selected plantations were sown in lines with line spacing of 2 m. Within each plot, four shrubs were randomly selected for sampling. Samples for each shrub were taken from two points (at 50 and 100 cm from the center of the shrub). The soil cores were collected using an 8-cm-diameter and 20-cm-length steel soil corer from surface down to 60 cm depth, which included 82% of root biomass (Zhang et al., 2018). The soil core samples were divided into three soil depths of 0–20, 20–40, and 40–60 cm. The soil samples for each depth were passed through a 0.45-mm sieve; root samples were placed into plastic bags and transported to an ice-filled cooler to the laboratory and stored at −4°C until later processing for fine root biomass estimate (a total of 120 sampling points and 360 samples). The root-free soils for each depth were retained separately for ingrowth cores method. The disturbance associated with installation of ingrowth cores bag may impact new root growth (Bauhus & Messier, 1999). To avoid this impact, in this study, the ingrown core method uses a labeling method (**Figure 1**). We cut the PVC pipe (outside diameters 90 mm, wall thickness 4.3 mm) into 5-cm-long rings, and each PVC ring was fix with three iron wires on the sampling point for marking, then root-free soils were filled back at the corresponding depth. One year later, in May 2019, the ingrowth cores were collected using an 8-cm-diameter steel soil corer, and the soil samples for each depth were passed through a 0.45-mm sieve, root samples transported to an ice-filled cooler to the laboratory and stored at -4°C until later processing for annual fine root production, mortality, and turnover rate (a total of 120 sampling points and 360 samples).

The soil water content and soil temperature in different depth (0–20, 20–40, and 40–60 cm) at different plantation ages were monitored continuously by ECH_2_O soil moisture monitoring system. As environmental factors, average monthly soil water content and soil temperature during the growing season from May to October were used. Soil bulk density (SBD) was estimated using the core method (a cylindrical metal 100 cm^3^ corer).

### 2.3 Laboratory analysis

In the laboratory, root samples obtained in 2018 were washed free of adhering soil, then discard roots larger than 2 mm in diameter. The fine roots (diameter ≤ 2 mm) were divided into two diameter classes (1 mm < diameter ≤ 2 mm and diameter ≤ 1 mm) and dried at 65°C to a constant mass, then weighed to the nearest 0.001 g to obtain fine root biomass. The ingrowth core root samples (obtained in 2019) were also washed free of adhering soil, and there were no roots larger than 2 mm in diameter. Therefore, the fine roots were divided into two diameter classes (1 mm < diameter ≤ 2 mm and diameter ≤ 1 mm) and then separated into living and dead roots based on morphological features (resilience, elasticity, and periderm color) (Brassard et al., 2009). We found that few of fine roots larger than 1 mm were found in the root samples, especially for dead roots, so this study only analyzed the fine roots smaller than 1mm (< 1mm in diameter). All root biomass was determined after oven-drying at 65°C till constant mass. Dried dead root samples were ground and sieved through a 100-mesh sieve, and the carbon concentrations were quantified using the induction furnace method with a CHNOS elemental analyzer. Soil organic carbon (SOC) was determined by potassium dichromate and sulfuric acid method. Soil total nitrogen (STN) was determined by Semimicro-Kjeldahl Method. Soil total phosphorus (STP) and soil total potassium (STK) were obtained with the HF-HCLO_4_^-^HNO_3_ digestion method using a 6300 ICP-AES (Forestry industry standard of the People's Republic of China, 1999). The samples were tested at the State Key Laboratory of vegetation and environmental change, Chinese Academy of Sciences.

### 2.4 Calculations and statistical analysis

Fine root production of *C. intermedia* plantations was calculated by balancing the living and dead fine root mass compartments according to the decision matrix method posed by Fairley and Alexander (1985). In the ingrowth core technique, fine root production was calculated as the sum of the live and dead root biomass (Sun et al., 2015). Fine root turnover rate (year^-1^) was calculated as the ratio of fine root production (g m^-2^ year^-1^) to fine root biomass (g m^-2^). The annual carbon input to SOC pool related to the fine roots was calculated by multiplying the annual fine root mortality by root carbon content (Huang et al., 2012).

The effects of plantation age and soil depth and their interactions on fine root biomass, production, mortality, and turnover rate of *C. intermedia* plantations were analyzed by two-way analysis of variance (ANOVA). Comparisons of the above parameters among five plantations and three soil depths were tested by one-way ANOVA and Duncan’s multiple range test. The relationship between fine root biomass, production, mortality, and turnover rate of *C. intermedia* plantation and plantation age was analyzed using a quadratic regression model. The relationship between fine root biomass, production, mortality, and turnover rate of *C. intermedia* plantation and environmental factors (SOC, STN, STP, STK, SBD, average monthly soil water content during the growing season from May to October, average monthly soil temperature during the growing season rom May to October) were evaluated by redundancy analysis (RDA). The statistical significance was tested by the Monte Carlo permutation method based on 999 runs with randomized data. All statistical analyses were conducted using SPSS 17.0 software and CANOCO 4.5 software.

## 3. Results

### 3.1 Fine root biomass, production, mortality, and turnover rate

**Figure 2:**
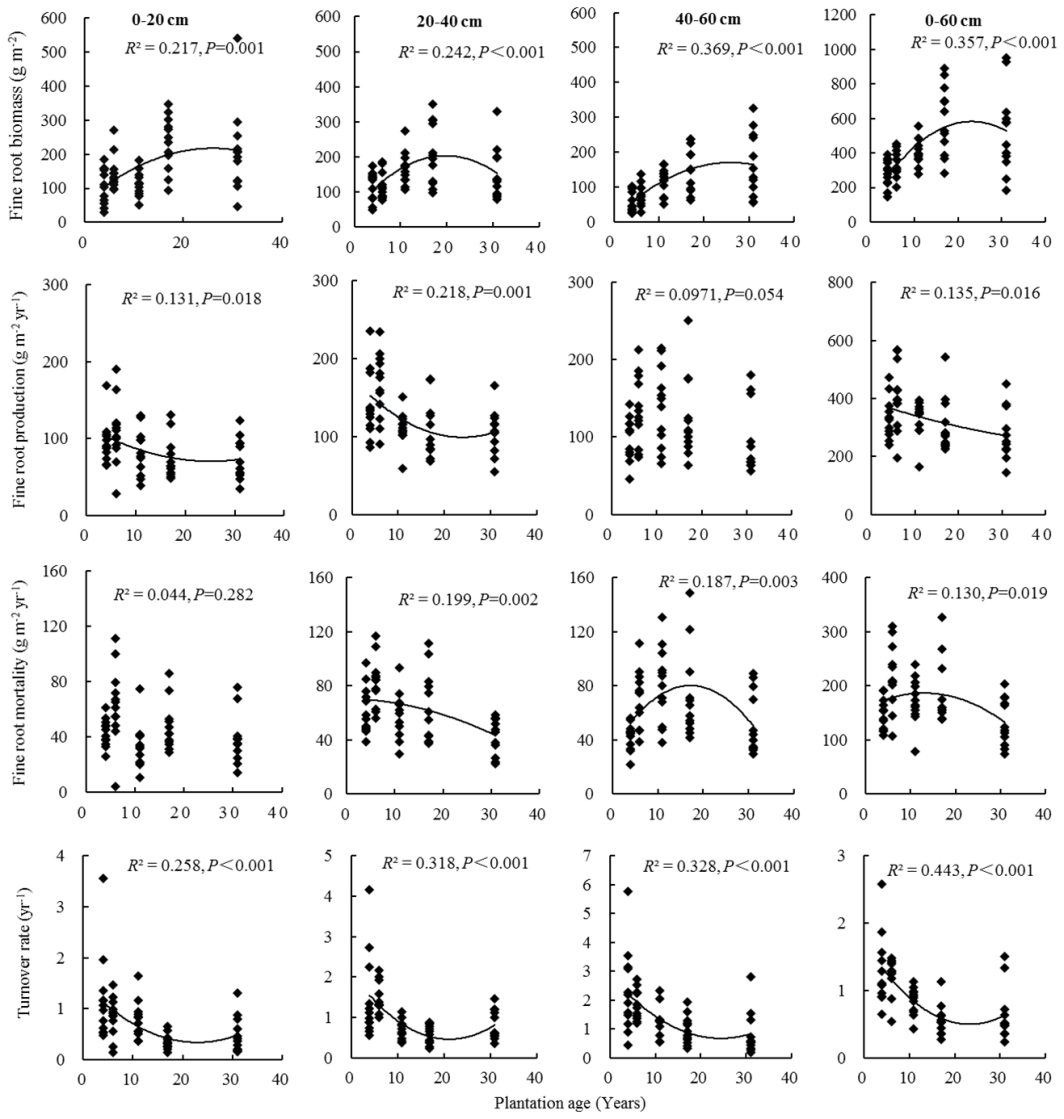
Fine root biomass, production, mortality, and turnover rate in relation to plantation age.

Fine root biomass, production, mortality, and turnover rate of *C. intermedia* plantation were significantly affected by both stand age and soil depth (*P* < 0.001) (**Table 1**). The quadratic regression analysis showed that the fine root biomass and turnover rate had a relatively higher fitting degree with the plantation age (**Figure 2**). At 0–60 cm depth, the fine root biomass, production, and mortality first increased and then decreased as the plantation age increased, and the fine root turnover rate first decreased and then slightly increased as the plantation age increased (**Table 2**). The fine root biomass peaked in 17-year-old plantation (692.16 ± 57.31 g m^-2^), the fine root production and mortality peaked in 6-year-old plantation (406.99 ± 32.53 and 217.36 ± 17.15 g m^-2^ year^-1^, respectively). The fine root turnover rates of 4- and 6-year-old plantations were significantly higher than other plantations (*P* < 0.05). At 0–20 cm and 20–40 cm depth, the trends of fine root biomass, production, and mortality changed with plantations age was in consistent with 0–60 cm depth. At 40–60 cm depth, the fine root biomass significantly increased as the plantation age increased (*P* < 0.05); the fine root production and mortality peaked in 11-year-old plantation.

**Table 1:** Effects of plantation age, soil depth, and their interactions on fine root biomass, production, mortality, and turnover rate.

	Plantation age		Soil depth		Plantation age × soil depth	
	F	P	F	P	F	P
Fine root biomass	15.867	< 0.001	9.236	< 0.001	1.781	0.084
Fine root production	6.867	< 0.001	18.326	< 0.001	2.024	0.047
Fine root mortality	10.064	< 0.001	15.067	< 0.001	3.237	0.002
Turnover rate	17.439	< 0.001	19.163	< 0.001	1.327	0.233

**Table 2:** Fine root biomass, production, mortality, and turnover rate of *C. intermedia* plantations (mean ± SE, *n* = 12).

	Depth (cm)	Plantation age (years)				
		4	6	11	17	31
Fine root biomass (g m^-2^)	0–20 cm	106.85 ± 15.11 Ab	144.51 ± 14.59 Ab	113.92 ± 10.53 Aa	232.66 ± 22.86 Bb	207.16 ± 36.31 Ba
	20–40 cm	118.02 ± 11.79 Ab	115.57 ± 11.03 Ab	167.11 ± 13.30 ABb	208.58 ± 24.99 Bab	151.56 ± 21.28 Aa
	40–60 cm	56.93 ± 8.69 Aa	76.77 ± 8.94 ABa	117.92 ± 10.94 BCa	150.92 ± 18.91 Ca	163.95 ± 26.32 Ca
	0–60 cm	281.79 ± 22.58 A	336.85 ± 22.04 A	398.95 ± 21.24 AB	592.16 ± 57.31 C	522.67 ± 69.33 BC
Fine root production (g m^-2^ year^-1^)	0–20 cm	94.90 ± 7.93 ABa	107.97 ± 11.88 Ba	82.47 ± 8.56 ABa	73.90 ± 7.68 Aa	73.25 ± 7.81 Aa
	20–40 cm	138.98 ± 12.34 BCb	164.20 ± 12.30 Cb	110.49 ± 6.03 ABab	108.09 ± 10.49 Ab	105.75 ± 8.23 Aa
	40–60 cm	98.75 ± 7.94 Aa	134.81 ± 12.87 Aab	138.39 ± 14.89 Ab	123.96 ± 15.14 Ab	94.91 ± 12.79 Aa
	0–60 cm	332.62 ± 20.14 A	406.99 ± 32.53 B	331.36 ± 17.99 A	305.96 ± 27.03 A	273.91 ± 25.26 A
Fine root mortality (g m^-2^ year^-1^)	0–20 cm	43.02 ± 2.84 Aa	63.49 ± 7.87 Ba	33.20 ± 4.71 Aa	47.04 ± 4.96 Aa	38.37 ± 5.12 Aa
	20–40 cm	61.96 ± 5.14 Bb	82.69 ± 5.21 Ca	58.78 ± 4.90 Bb	64.14 ± 7.53 Ba	43.18 ± 3.87 Aa
	40–60 cm	42.31 ± 2.85 Aa	71.19 ± 6.03 BCa	80.74 ± 7.94 Cc	72.19 ± 9.47 BCa	51.77 ± 6.57 ABa
	0–60 cm	147.29 ± 8.41 AB	217.36 ± 17.15 C	172.72 ± 12.06 AB	183.37 ± 17.29 BC	133.32 ± 12.44 A
Turnover rate (year^-1^)	0–20 cm	1.18 ± 0.25 Ca	0.84 ± 0.11 BCa	0.78 ± 0.10 BCa	0.35 ± 0.04 Aa	0.49 ± 0.10 ABa
	20–40 cm	1.47 ± 0.31 Bab	1.48 ± 0.11 Bb	0.71 ± 0.07 Aa	0.59 ± 0.07 Aa	0.81 ± 0.10 Aa
	40–60 cm	2.30 ± 0.42 Cb	1.87 ± 0.15 BCc	1.26 ± 0.15 ABb	0.96 ± 0.14 Ab	0.83 ± 0.21 Aa
	0–60 cm	1.29 ± 0.15 B	1.23 ± 0.08 B	0.85 ± 0.06 A	0.57 ± 0.07 A	0.64 ± 0.11 A

Different uppercase letters following values indicate a significant difference among plantation ages; different lowercase letters following values indicate a significant difference among soil depths, according to Duncan’s multiple range test (P < 0.05), n = 12.

Fine root biomass, production, mortality, and turnover rate of different age plantations changed with soil depth were inconsistent. In 4- and 6-year-old plantations, fine root biomass at 0–20 cm and 20–40 cm depth were significantly higher than that of 40–60 cm depth (*P* < 0.05); fine root production and mortality first increased and then decreased as soil depth increased, peaked at 20–40 cm depth. In 11-year-old plantation, fine root biomass at 20–40 cm depth was significantly higher than that of 0–20 cm and 40–60 cm depth (*P* < 0.05); fine root production and mortality significantly increased as soil depth increased (*P* < 0.05). In 17-year-old plantation, fine root biomass significantly decreased as soil depth increased (*P* < 0.05); fine root production and mortality increased as soil depth increased. There was no significant difference in fine root biomass, productivity, mortality, and turnover rate of 31-year-old plantation with the increase of soil depth (*P* > 0.05). Fine root turnover rate of 4-, 6-, 11-, and 17-year-old plantations significantly increased as soil depth increased (*P* < 0.05).

### 3.2 Relationship between fine root biomass, annual fine root production, mortality, turnover rate, and environmental factors

**Figure 3:**
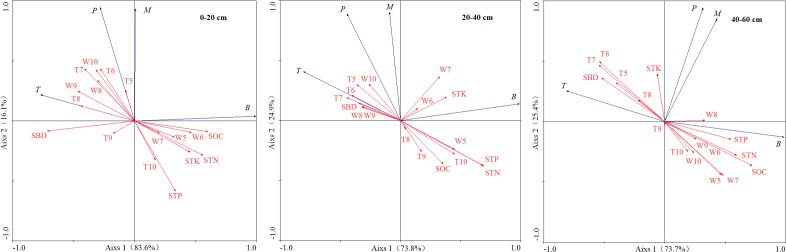
Ordination diagram of RDA on fine root biomass, production, mortality and turnover rate with environmental factors at different soil depths. B, fine root biomass; M, fine root mortality; P, fine root production; T, fine root turnover rate; SOC, soil organic carbon; STN, soil total nitrogen; STP, soil total phosphorus; STK, soil total potassium; SBD, soil bulk density; W5-W10, average monthly soil water content during the growing season from May to October; T5-T10, average monthly soil temperature during the growing season from May to October.

The relationship between fine root biomass, production, mortality, turnover rate, and environmental factors at different depths was assessed through RDA (**Figure 3**). At depth of 0–20 cm, fine root biomass had a significant negative correlation with SBD and positive correlation with SOC and STN content. Fine root production had a significant negative correlation with STP and positive correlation with soil temperature of July and June. Fine root mortality had a negative correlation with STP. Fine root turnover rate had a positive correlation with soil water content of September.

At depth of 20–40 cm, fine root biomass had a positive correlation with STN and STP content, fine root production and turnover rate had a significant negative correlation with STP and STN content, and fine root mortality had a negative correlation with SOC.

At depth of 40–60 cm, fine root biomass had a significant positive correlation with SOC, STN, and STP content, soil water content of May and July, and negative correlation with soil temperature of June and July and SBD. Fine root turnover rate had a significant negative correlation with STP, STN and SOC content, soil water content of May, and positive correlation with soil temperature of June and July and SBD. There was no significant correlation between fine root production, mortality, and environmental factors.

### 3.3 Contribution of fine root mortality to SOC stocks

**Figure 4:**
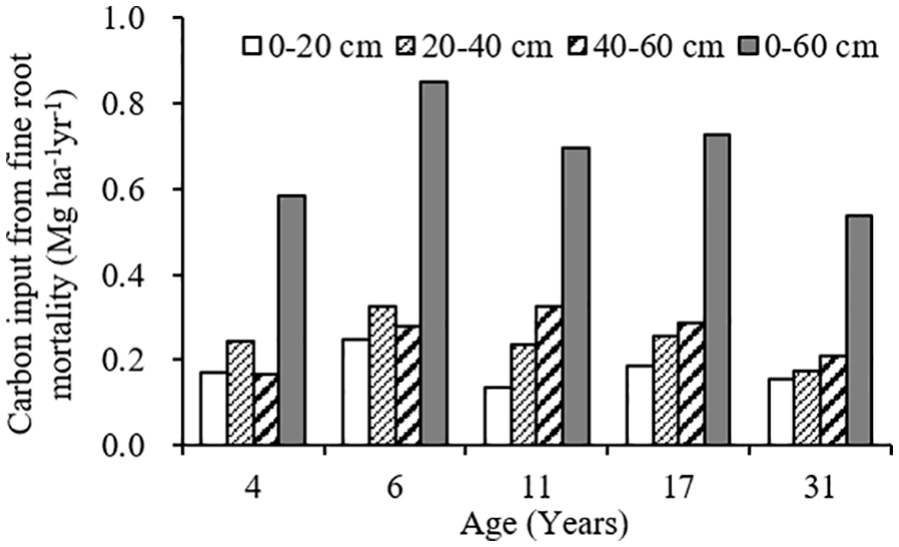
Carbon input from fine root mortality at different soil depths of *C. intermedia* plantations of different ages.

**Figure 5:**
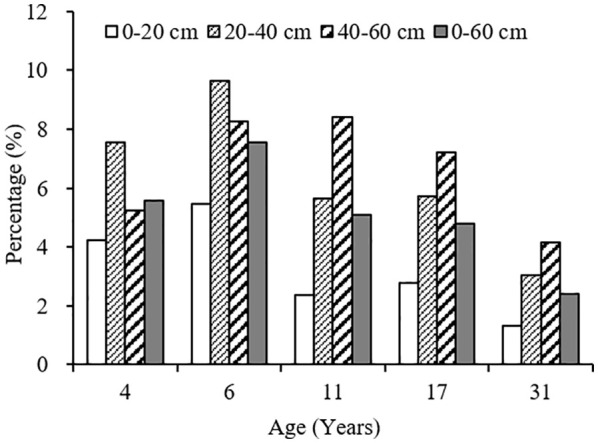
The percentage of carbon input from fine root mortality account for the SOC stocks at different soil depths of *C. intermedia* plantations of different ages.

The variation range of carbon input by fine root mortality across different plantation age at 0–60 cm soil depth was 0.54–0.85 Mg ha^-1^year^-1^, accounting for 2.40–7.54% of the SOC stocks, first increased, and then decreased as the plantation age increased, peaked in 6-year-old plantation (**Figures 4**, **5**). The percentage of carbon input from fine root mortality accounting for the SOC stocks of 31-year-old plantation was obviously lower than other plantations. Comparing the three soil depths, carbon input from fine root mortality of 4- and 6-year-old plantations at 20–40 cm soil depths were more than other soil depths, while carbon input from fine root mortality of 11-, 17-, and 31-year-old plantations increased as the soil depth increased. The percentage of carbon input from fine root mortality accounting for the SOC stocks of the three soil depths have the same trend.

## 4. Discussion

### 4.1 Fine root dynamics

The fine root biomass (except for 4-year-old plantation) and production for *C. intermedia* plantation were higher than the average fine root biomass (332 g m^-2^) and production (250 g m^-2^ year^-1^) (< 2 mm in diameter) for European forests (Neumann et al., 2019). Fine root biomass for 17- and 31-year-old plantation were similarly to the fine root biomass (< 2 mm in diameter) for boreal forest (5.28 Mg ha^−1^, *n* = 765), and fine root production for 4-, 6-, and 17-year-old plantations were higher than fine root production (< 2 mm in diameter) for the boreal forest (2.83 Mg ha^−1^year^-1^) (Yuan and Chen, 2010). The fine root biomass and production were also higher than *Salix psammophila* (189.96* g* m^-2^ and 310.22 g m^-2^ year^-1^), *Hedysarum mongolicum* (21.58* g* m^-2^ and 37.54 g m^-2^ year^-1^), and *Artemisia ordosica* (41.09* g* m^-2^ and 35.48 g m^-2^ year^-1^) (fine root < 2 mm in diameter) in northwest China (Lai et al., 2016). Our result reveals that the fine root biomass and production (< 1mm in diameter) of *C. intermedia* plantation in alpine sandy land were similarly or higher to some forest, it has a strong carbon sequestration potential.

Fine root biomass, production, and mortality first increased and then decreased as the development of plantation. Plantations with lower age have higher fine root production, mortality, and turnover rate. All these results indicating that fine roots in young stands had a faster metabolism. Finér et al. (2011) also reported that fine root turnover rate declined with stand age in their meta-analyses. With the development of plantations, how fine root biomass changes and when it reaches to peak may depend on ecosystem types (Yuan and Chen, 2010). Mund et al. (2002) reported that, along a chronosequence of spruce forest in central Europe, fine root (< 2 mm) biomass increased from 16- to 112-year-old stands and then decreased at an age of 142 years. In Southern Ontario, Canada, with the development of four white pine plantations (2-, 15-, 30-, and 65-year-old), fine root biomass firstly increased with stand age and reached to a peak in the 30-year-old stand (6.2 t ha^-1^), then it decreased in the 65-year-old stand (3.5 t ha^-1^) (Peichl and Arain, 2007).

With the *C. intermedia* plantation development, fine roots biomass decreased with the soil depth increased; conversely, fine root production and mortality increased with the soil depth increased. Lai et al. (2016) reported that fine-root production of *S. psammophila* was mostly concentrated in the 0–20 cm depth interval; *H. mongolicum* and *A. ordosica* in all soil depths were evenly distributed in northwest China. Some studies found that the highest amount of necromass occurred close to surface soil, and the dead fine roots mass decreased with soil depths (Wang et al., 2015; Lai et al., 2016). Wang et al. (2015) reported that the live and dead fine roots mass of *Pinus koraiensis* forest dramatically decreased with soil depths in northeastern China. The differences in vertical distribution of fine roots production and mortality among studies suggested that different species might have different adaption mechanisms to their environment, especially when resources are limited. Some studies revealed that the distribution patterns of fine root production in different soil profiles were different due to heterogeneity of soil nutrients and water (Wang et al., 2014). In this study, the fine roots in deep soil were more active and had a faster metabolism.

### 4.2 The relationship between fine root dynamics and environmental factors

Fine root production and mortality were negative correlated with soil nutrients (especially STP) at depth of 0–20 and 20–40 cm. Leguminous plants need a large amount of phosphorus to form or maintain a symbiotic system, and rhizobium nitrogenase also needs a large amount of phosphorus in the nitrogen fixation process (Vitousek et al., 2002). Inagaki et al, 2009 reported that legume species demand more P than non-legume species. *C. intermedia* is a leguminous shrub, and the fine root dynamics may be more susceptible to the influence of STP content. The cost-benefit hypothesis states that favorable conditions increase fine root life span, whereas stress reduces fine root life span (McCormack and Guo, 2014). Lack of soil fertility in desert ecosystems may cause plants to allocate more carbon to root systems and form a denser fine root network (Loiola et al., 2016). However, some studies have shown that N addition had no effect on root biomass (Wang et al., 2020). In the early period of vegetation restoration, with increasing aboveground production, litter accumulated resulting in a higher nutrient in surface soil (Wang et al., 2018a), fine roots in sub-soil accelerated metabolism for absorbing limited nutrients. With the plantation age increased, the nutrient conditions of the sub-surface soil were also improved (Li et al., 2019), fine roots in deeper soil accelerated metabolism for absorbing limited nutrients, resulting the fine roots active soil layer transitions to the deeper soil layer with the plantation development. Our result indicating that, in the cold, arid and barren sandy land ecosystem, fine root production, and mortality were greater, and metabolism was faster in lower soil nutrients environment.

At depth of 0–20 cm, fine root production was positive correlated with soil temperature of July and June. The temperature in July and June in the study area is relatively high, and the plant growth is more vigorous at this time. Surface soil is more susceptible to temperature, and the soil temperature increases can promote the fine root production (Wang et al., 2014). Wang et al. (2015) reported that, in a mixed mature *Pinus koraiensis* forest in northeastern China, monthly fine root production was associated with current month air temperature.

### 4.3 SOC input from fine root mortality

Fine roots not only store a large amount of organic carbon but also transfer organic carbon from dead roots to the soil by fine root turnover. Therefore, fine root production and turnover are an important part of carbon and nutrient cycles in terrestrial ecosystems (Gill and Jackson, 2000). Many studies have reported the importance of fine root mortality for SOC in forest soils (Clemmensen et al., 2013; Prietzel & Christophel, 2014; Wang et al., 2018b). In the arid ecosystems of Xinjiang, China, SOC input from fine root mortality at 0–60 cm soil depth was 42. 68 g·m^–2^·year ^–1^ for *Tamarix ramosissima* community; the percentage of SOC input from fine root mortality account for the SOC stocks was 2.12% (Wang et al., 2014). In this study, the variation range of carbon input from fine root mortality at 0–60 cm soil depth was 0.54–0.85 Mg ha^-1^year^-1^ for different age *C. intermedia* plantation, accounting for 2.40–7.54% of the SOC stocks. Although the SOC input from fine root mortality only accounts for a small part of the SOC stocks, the fine root is more conducive to the long-term accumulation of SOC in desert ecosystems.

With the development of plantation, carbon input to soil from fine root mortality first increased and then decreased, and increased as the soil depth increased. Some studies reported that fine root mortality was higher at surface soil, and decreased with soil depths (Wang et al., 2015; Lai et al., 2016; Du et al., 2019). The contribution of fine root mortality to SOC stocks will be significantly underestimated if we calculate the carbon input to soil only using the fine root mortality at surface soil, especially in mature plantations (11-, 17-, and 31-year-old plantations). Therefore, we suggest that the influences of plantation age and soil depth should be taken into account when calculating the contribution of fine root mortality to SOC stocks.

## 5. Conclusions

In *C. intermedia* plantation in alpine sandy land, the fine root biomass and production were similarly or higher to some forest; it has a strong carbon sequestration potential. Fine root production, mortality, and turnover rate were higher in young stands. With the development of plantation, the fine roots in deep soil were more active and had a faster metabolism. Fine roots regenerate faster in lower soil nutrients environment. The soil temperature increases could promote the fine root production in surface soil. The variation range of carbon input by fine root mortality across different plantation age at 0–60 cm soil depth was 0.54–0.85 Mg ha^-1^year^-1^, accounting for 2.40–7.54% of the SOC stocks. The fine root is more conducive to the long-term accumulation of SOC in desert ecosystems.

## Data availability statement

The raw data supporting the conclusions of this article will be made available by the authors, without undue reservation.

## Author contributions

ZJ and QL designed the study and provided primary funding. QL, LH, XZ and HY performed field sampling and measurements. QL and ZJ wrote the draft manuscript and analyzed the data. LH, XZ and HY provided editorial advices. All authors contributed to the article and approved the submitted version.

## References

[B1] Batkhuu N. O. Byambadorj S. O. Park B. B. Terzaghi M. Scippa G. S. Stanturf J. A.. (2021). Root biomass distribution of *Populus sibirica* and *Ulmus pumila* afforestation stands is affected by watering regimes and fertilization in the Mongolian semi-arid steppe. Front. Plant Sci. 12, 638828. doi: 10.3389/fpls.2021.638828 33968099 PMC8102691

[B2] Bauhus J. Messier C. (1999). Soil exploitation strategies of fine roots in different tree species of the southern boreal forest of eastern Canada. Can. J. Forest. Res. 29 (2), 260–273. doi: 10.1139/x98-206

[B3] Brassard B. W. Chen H. Y. H. Bergeron Y. (2009). Influence of environmental variability on root dynamics in northern forests. Crit. Rev. Plant Sci. 28, 179–197. doi: 10.1080/07352680902776572

[B4] Clemmensen K. E. Bahr A. Ovaskainen O. Dahlberg A. Ekblad A. Wallander H.. (2013). Roots and associated fungi drive long-term carbon sequestration. Science 339, 1615–1618. doi: 10.1126/science.1231923 23539604

[B5] Du H. Lu L. Liang S. Zeng F. Wang K. Peng W.. (2019). Seasonal changes and vertical distribution of fine root biomass during vegetation restoration in a karst area, southwest china. Front. Plant Sci. 9, 2001. doi: 10.3389/fpls.2018.02001 30687380 PMC6337902

[B6] Fairley R. I. Alexander I. J. (1985). “Methods of calculating fine root production in forests,” in Ecological interactions in soil: Plants, microbes and animals. Eds. Fitter A. H. Atkinson D. Read D. J. (Oxford, UK: Blackwell Scientific Publications), 37–42.

[B7] Finér L. Ohashi M. Noguchi K. Hirano Y. (2011). Fine root production and turnover in forest ecosystems in relation to stand and environmental characteristics. For. Ecol. Manage. 262, 2008–2023. doi: 10.1016/j.foreco.2011.08.042

[B8] Forestry industry standard of the People's Republic of China (1999). Forest soil analysis method (Beijing: China Standard Press), 1–108.

[B9] Gill R. A. Jackson R. B. (2000). Global patterns of root turnover for terrestrial ecosystems. New Phytol. 147, 13–31. doi: 10.1046/j.1469-8137.2000.00681.x

[B10] Huang G. Zhao X. Y. Li Y. Q. Cui J. Y. (2012). Restoration of shrub communities elevates organic carbon in arid soils of northwestern China. Soil Biol. Biochem. 47, 23–132. doi: 10.1016/j.soilbio.2011.12.025

[B11] Inagaki M. Inagaki Y. Kamo K. Titin J. (2009). Fine−root production in response to nutrient application at three forest plantations in sabah, Malaysia: higher nitrogen and phosphorus demand by acacia mangium. J. For. Res. 14 (3), 178–182. doi: 10.1007/s10310-009-0113-0

[B12] Ji Z. J. Yuan X. X. Mu S. M. L. Zhang J. X. Wang X. Y. (2019). The biogeographic distribution of the *Caragana rhizobia* . J. Inner Mongolia Univ. Nationalities 34 (5), 421–423, 460. doi: 10.14045/j.cnki.15-1220.2019.05.010

[B13] Jiang X. Y. Jia X. Gao S. G. Jiang Y. Wei N. N. Han C.. (2021). Plant nutrient contents rather than physical traits are coordinated between leaves and roots in a desert shrubland. Front. Plant Sci. 12, 734775. doi: 10.3389/fpls.2021.734775 34764966 PMC8576145

[B14] Lai Z. Liu J. Zhang Y. Wu B. Qin S. Sun Y.. (2017). Introducing a shrub species in a degraded steppe shifts fine root dynamics and soil organic carbon accumulations, in northwest China. Ecol. Eng. 100, 277–285. doi: 10.1016/j.ecoleng.2017.01.001

[B15] Lai Z. Zhang Y. Liu J. Wu B. Qin S. Fa K. (2016). Fine-root distribution, production, decomposition, and effect on soil organic carbon of three revegetation shrub species in northwest China. For. Ecol. Manage. 359, 381–388. doi: 10.1016/j.foreco.2015.04.025

[B16] Li Q. X. Yang D. F. Jia Z. Q. Zhang L. H. Zhang Y. Y. Feng L. L.. (2019). Changes in soil organic carbon and total nitrogen stocks along a chronosequence of *caragana intermedia* plantations in alpine sandy land. Ecol. Eng. 133, 53–59. doi: 10.1016/j.ecoleng.2019.03.003

[B17] Liu X. P. Luo Y. G. Cheng L. Hu H. J. Wang Y. H. Du Z. (2021). Effect of root and mycelia on fine root decomposition and release of carbon and nitrogen under *Artemisia halodendron* in a semi-arid sandy grassland in China. Front. Plant Sci. 12,698054. doi: 10.3389/fpls.2021.698054 34539692 PMC8442746

[B18] Loiola P. P. Carvalho G. H. Batalha M. A. (2016). Disentangling the roles of resource availability and disturbance in fine and coarse root biomass in savannah. Austral. Ecol. 41, 255–262. doi: 10.1111/aec.12306

[B19] Majdi H. Truus L. Johansson U. Nylund J. E. Wallander H. (2008). Effects of slash retention and wood ash addition on fine root biomass and production and fungal mycelium in a Norway spruce stand in SW Sweden. For. Ecol. Manage. 255 (7), 2109–2117. doi: 10.1016/j.foreco.2007.12.017

[B20] McCormack M. L. Eissenstat D. M. Prasad A. M. Smithwick E. A. H. (2013). Regional scale patterns of fine root lifespan and turnover under current and future climate. Glob Change Biol. 19 (6), 1697–1708. doi: 10.1111/gcb.12163 23504802

[B21] McCormack M. L. Guo D. (2014). Impacts of environmental factors on fine root lifespan. Front. Plant Sci. 5, 205. doi: 10.3389/fpls.2014.00205 24904605 PMC4032987

[B22] Mund M. Kummetz E. Hein M. Bauer G. A. Schulze E. D. (2002). Growth and carbon stocks of a spruce forest chronosequence in central Europe. For. Ecol. Manage. 171, 275–296. doi: 10.1016/S0378-1127(01)00788-5

[B23] Neumann M. Godbold D. L. Hirano Y. Finér L. (2019). Improving models of fine root carbon stocks and fluxes in European forests. J Ecol 108 (2), 496–514. 10.1111/1365-2745.13328 PMC7065197 32189723

[B24] Nie X. Wang D. Chen Y. Yang L. Zhou G. (2022). Storage, distribution, and associated controlling factors of soil total phosphorus across the northeastern Tibetan plateau shrublands. J. Soil Sci. Plant Nutr. 22, 2933–2942. doi: 10.1007/s42729-022-00857-1 PMC9039665 35498684

[B25] Peichl M. Arain M. A. (2007). Allometry and partitioning of above- and belowground tree biomass in an age-sequence of white pine forests. For. Ecol. Manage. 253, 68–80. doi: 10.1016/j.foreco.2007.07.003

[B26] Prietzel J. Christophel D. (2014). Organic carbon stocks in forest soils of the German Alps. Geoderma 221–222, 28–39. doi: 10.1016/j.geoderma.2014.01.021

[B27] Ruess R. W. Hendrick R. L. Burton A. J. Pregitzer K. S. Sveinbjornsson B. Allen M. E.. (2003). Coupling fine root dynamics with ecosystem carbon cycling in black spruce forests of interior Alaska. Ecol. Monogr. 73, 643–662. doi: 10.1890/02-4032

[B28] Song Z. P. Wang X. M. Liu Y. H. Luo Y. Q. Li Z. L. (2022).Allocation strategies of carbon, nitrogen, and phosphorus at species and community levels with recovery after wildfire. Front. Plant Sci. 13, 850353. doi: 10.3389/fpls.2022.850353 35481138 PMC9037545

[B29] Sun T. Dong L. L. Mao Z. J. Li Y. Y. (2015). Fine root dynamics of trees and understorey vegetation in a chronosequence of betula platyphylla stands. For. Ecol. Manage. 346, 1–9. doi: 10.1016/j.foreco.2015.02.035

[B30] Vitousek P. M. Hättenschwiler S. Olander L. Allison S. (2002). Nitrogen and nature. AMBIO: A J. Hum. Environ. 31 (2), 97–101. doi: 10.1579/0044-7447-31.2.97 12078015

[B31] Wang D. Chi Z. Yue B. Huang X. Liu Y. (2020). Effects of mowing and nitrogen addition on the ecosystem c and n pools in a temperate steppe: a case study from northern china. Catena 185, 104332. doi: 10.1016/j.catena.2019.104332

[B32] Wang C. G. Han S. J. Zhou Y. M. Zhang J. H. Zheng X. B. Dai G. H.. (2015). Fine root growth and contribution to soil carbon in a mixed mature *pinus koraiensis* forest. Plant Soil 400, 275–284. doi: 10.1007/s11104-015-2724-x

[B33] Wang D. Zhang B. Zhu L. Yang Y. Li M. (2018a). Soil and vegetation development along a 10-year restoration chronosequence in tailing dams in the xiaoqinling gold region of central china. Catena 167, 250–256. doi: 10.1016/j.catena.2018.05.007

[B34] Wang C. G. Zhao C. Brunner I. Zhen Z. Zhu X. Li J.. (2018b). Global patterns of dead fine root stocks in forest ecosystems. J. Biogeogr. 45, 1378–1394. doi: 10.1111/jbi.13206

[B35] Wang J. Zhao X. C. Lai L. M. Zhu L. H. Wang Y. J. Zhou J. H.. (2014). Contribution of fine root production and turnover to soil organic carbon in *Tamarix ramosissima* community in sangong river basin of xinjiang, China. For. Res. 27 (6), 809–814. doi: 10.13275/j.cnki.lykxyj.2014.06.016

[B36] Yuan Z. Y. Chen H. (2010). Fine root biomass, production, turnover rates, and nutrient contents in Boreal forest ecosystems in relation to species, climate, fertility, and stand age: Literature review and meta-analyses. Crit. Rev. Plant Sci. 29 (4), 204–221. doi: 10.1080/07352689.2010.483579

[B37] Yuan Z. Y. Chen H. Y. H. (2012). Fine root dynamics with stand development in the boreal forest. Funct. Ecol. 26 (4), 991–998. doi: 10.1111/j.1365-2435.2012.02007.x

[B38] Zhang L. H. Wang X. Q. Jia Z. Q. Li Q. X. Chen X. J. (2018). Root distribution characteristics of *Caragana intermedia* plantations at different ages in alpine sandy land. J. Arid Land Resour. Environ. 32 (11), 163–168. doi: 10.13448/j.cnki.jalre.2018.350

